# Implementing a 2019 coronavirus disease airway management strategy for a provincial critical care and ground transport program

**DOI:** 10.1017/cem.2020.413

**Published:** 2020-06-03

**Authors:** Scott MacDonald, George Kovacs, Tobias Witter, Yves Leroux, Steven Crocker, Lindsay Richards

**Affiliations:** * Department of Emergency Medicine, Dalhousie University, Halifax, NS; † Department of Critical Care, Department of Anesthesia, Pain Management, and Perioperative Medicine, Dalhousie University, Halifax, NS; ‡ Division of Emergency Medical Services, Department of Emergency Medicine, Dalhousie University, Halifax, NS; § Critical Care Transport Program, EHS LifeFlight, Enfield, NS

**Keywords:** Airway, COVID-19, critical care transport, emergency medical services, pandemic

The 2019 coronavirus disease (COVID-19) pandemic has challenged our healthcare system on numerous fronts. The complexity and volume of critically ill patients pushed systems in China, Italy, Spain, and, more recently, the United States, particularly the state of New York, to the point of crisis as providers scrambled to manage this new disease. Based on modelling from these “hot spots” and other areas around the world, provincial healthcare systems across the country launched into preparation mode to potentially manage volumes of patients who could similarly threaten capacity.

With each passing day as these surges rolled through areas around the world, we learned a little more about the natural history of this disease and how to manage the patients at various stages of illness. In Canada, our public health and political leaders have played their roles admirably in prevention and protection, and, while we will measure their successes in hindsight, we are sensing that their efforts have altered the course of the pandemic for the Canadian experience.

As COVID-19 hit New York with a vengeance combined with 24/7 worst-case scenario messaging in the media, there was a palpable sense of fear in our communities and amongst healthcare providers anticipating what was to come. Provincial health leaders countered with a 24/7 work ethic, ramping up operations and planning with coordinated efforts to build capacity, as well as protocolize care pathways to keep ahead of the curve. What many of us have witnessed is unprecedented collaboration, the removal of silos of care, and a willingness to adapt.

The focus of most planning efforts, again based on lessons learned from elsewhere, was on safely caring for potentially large volumes of patients needing access to critical care. “The risk of respiratory failure requiring critical care support in patients infected with COVID-19 is significant.”^1^ Acute care provider teams in prehospital and hospital settings had to prepare for the arrival, management, and transfer of care of these patients. Both the patient and provider teams are at risk while caring for the sickest of COVID-19 cases. While patient safety continues to be a goal, what is different during this pandemic is that attention to the safety of the staff caring for the patient assumes equal or, indeed, more significant importance.

In Nova Scotia (NS), a small but mighty province with a single prehospital provider, Emergency Health Services (EHS), swiftly moved while consulting with other providers from across the country. Sharing the knowledge and expertise of clinical leaders in prehospital care, emergency medicine, anesthesiology, and critical care medicine, we aligned with operational and governmental counterparts to collaborate, plan, and execute a cohesive COVID-19 strategy.

As an example, the NS provincial strategy for airway management for COVID-19 patients highlights a combined effort of many moving parts culminating in a cohesive plan to manage and transport the sickest patients in the province. An effort to standardize an approach to airway management in emergency departments quickly grew into a provincial strategy for all airway providers. The “Airway Management Guidelines for Patients with Known or Suspected COVID-19 Infection” was generated to provide the best available evidence.^2^ The recommendations were supported by “Level C” evidence (low quality, expert opinion, and consensus). Context-sensitive processes around personal protective equipment (PPE), safe oxygen delivery methods, and advanced airway management needed to be developed, disseminated, and monitored in real-time.

The Nova Scotia Health Authority “Airway Management Guidelines for Patients with Known or Suspected COVID-19 Infection” documents (three iterations) were shared, and EHS clinical leadership used the recommendations to guide prehospital airway pathways. The highlights from the guidance document regarding changes in prehospital approach to COVID-19 patients include^2^:

1. Emphasizing provider safety as the priority in managing acute illness related to COVID-19, including having access to and training for safe PPE use
2. The use of a specific oxygenation escalation plan for the different types of potential COVID-19 patient encounters
3. Guidance for bridging oxygenation strategies using available or newly deployed equipment (i.e., viral-filtered continuous positive airway pressure [CPAP])
4. Granular detail on performing rapid sequence intubation (RSI) as a first approach to securing the airway
5. Special attention to alternative, safe means of maintaining oxygenation and re-oxygenation throughout airway management
6. Improving access to and skills by using video laryngoscopy (VL) to achieve safe, high first-pass success intubations
7. Provision of detailed, accessible educational materials to support recommendations made in the provincial document

EHS LifeFlight (NS's Critical Care Transport Program) clinical practice guidelines (CPGs) are evidence-based and modelled on a standard of care similar to hospital-based critical care but adapted to transport medicine. LifeFlight had to ensure that they could safely apply these hospital-based recommendations around airway management for COVID-19 patients.

EHS, working closely with other clinical leaders within the health authority, used this resource document as a springboard to inform changes to usual practice related to COVID-19. Over 90% of transported LifeFlight missions involve critically ill patients with time-sensitive conditions or requiring escalation in critical care. In 10% of cases, LifeFlight team members are the primary airway providers on scene or at the sending facility. The following time-sensitive issues needed addressing to ensure a safe and effective COVID-19 airway management strategy:

1. Acquisition of new equipment
2. Modification of existing relevant standard operating procedures and CPGs
3. Skills training and simulation
4. Close monitoring and feedback throughout training and implementation of the airway management strategy
5. PPE for advanced airway management trialed and secured for our provincial transport program with a regular training schedule for safe PPE practice

Recommendations from the Nova Scotia Health Authority airway management document supported the use of an “indirect” Macintosh blade VL device and encouraged routine use of a bougie as a means of safely achieving a high first-pass intubation success rate. Working with the government and EHS, successful sourcing, and purchasing of a new VL system happened quickly.

The acquired Storz CMAC® VL met the requirements of our transport program and is supported by good evidence as a device to help us safely maintain our high first-pass success goals for intubation (> 90%).^3^ The standard approach pre-pandemic for LifeFlight was direct laryngoscopy aided by bougie (75%), with a first-pass success > 95%. While the approach described in the airway guideline was not a new concept for LifeFlight, the use of the new equipment and first approach was a departure from usual practice.

Introducing new equipment and a change in the first approach made access to appropriate training an urgent need. In addition to traditional simulation using manikins, Dalhousie University graciously responded by granting special permission to access clinical cadavers through the Human Body Donation Program, despite the university being closed for traditional educational programming. Training with the clinical cadavers, the adult and pediatric critical care teams were ready to operationalize the new equipment within days of procurement.

The EHS prehospital ground system additionally adjusted its airway management strategy for patients not requiring critical care transport or for whom LifeFlight cannot support promptly.

**Figure 1.**
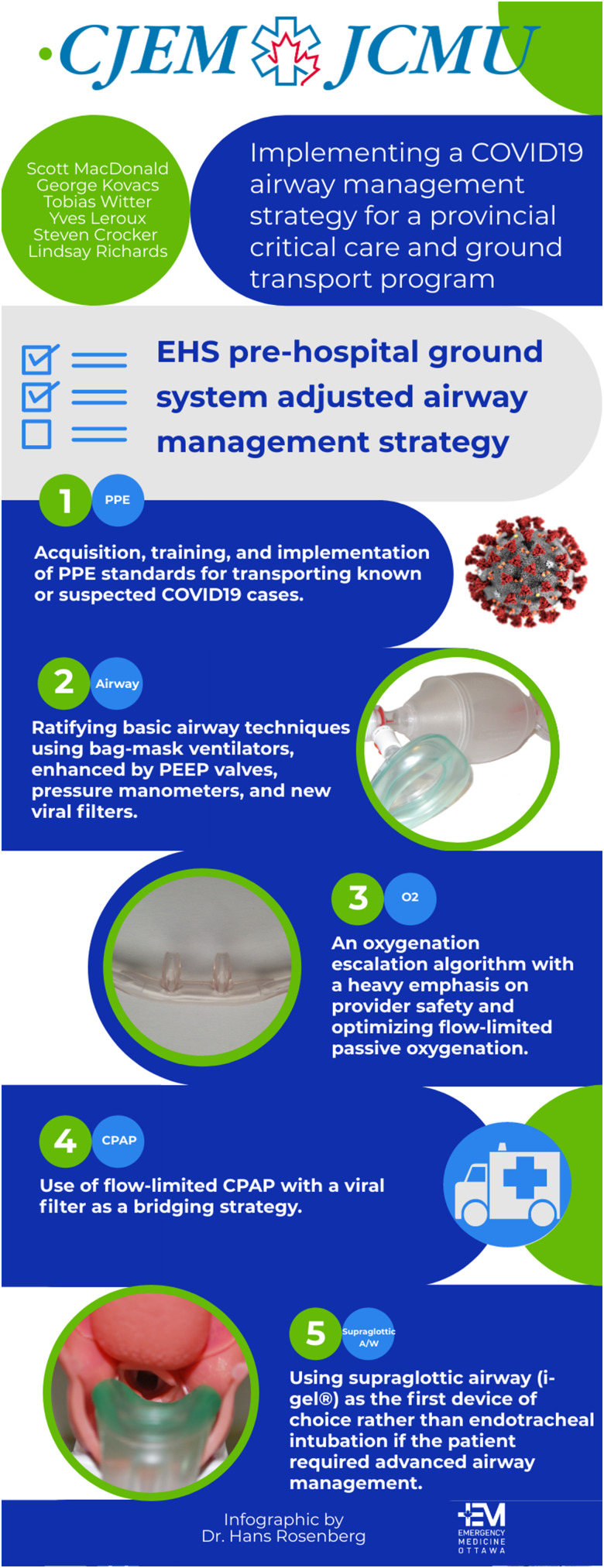
COVID-19 airway management strategies for patient transport.

Changes included (Figure 1):

1. Acquisition, training, and implementation of PPE standards for transporting known or suspected COVID-19 cases
2. Ratification of basic airway techniques using bag-mask ventilators, enhanced by positive end expiratory pressure valves, pressure manometers, and new viral filters
3. An oxygenation escalation algorithm with a heavy emphasis on provider safety and optimizing flow-limited passive oxygenation
4. Use of flow-limited CPAP with a viral filter as a bridging strategy
5. Use of supraglottic airway (i-gel®) as the first device of choice rather than endotracheal intubation if the patient requires advanced airway management

In NS, endotracheal intubation by direct laryngoscopy is within the scope of practice for Advanced Care Paramedics, but RSI is not. In a non-cardiac arrest patient, it was decided that attempting intubation without neuromuscular blockade or access to a VL would put providers at increased risk of exposure. For advanced airway management in cardiac arrest patients, a supraglottic airway (i-gel®) was prioritized. This decision was supported by recently updated American Heart Association guidelines based on historical system intubation success rates.^4^

Managing and moving COVID-19 patients have caused unprecedented stress on our healthcare system and the individuals working within it. The quick action and collaboration to implement a new airway management strategy for NS prehospital providers demonstrate a tangible success in the adaptability of an entire system to maximize opportunities to act. In the process, COVID-19 has exposed gaps that predate the pandemic. How does our prehospital system support patients needing transport when our critical care teams are not available? Do we expand the scope of practice for a cohort of prehospital providers? Do we augment the prehospital system with hospital-based transport providers? Do we understand the real risks of responding to a surge in a system near or over capacity? We have been successful in closing some of the gaps through collaboration and ingenuity, but continuing the call to action and answering these and other questions will hopefully be the upside legacy of this pandemic.
